# Large Posterior Communicating Artery Aneurysm Presenting as Hemiparesis: A Case Report

**DOI:** 10.7759/cureus.77270

**Published:** 2025-01-11

**Authors:** Fardous Abbasher, Noman Shah, Muhammad Mohsin Khan, Sama Al-Dori, Amr Rida El Mohamad, Abdullah Illeyyan, Omar M Shihadeh, Ali Sulaiman, Muath Hussein, Ali Ayyad

**Affiliations:** 1 Neurosurgery, Hamad General Hospital, Doha, QAT; 2 Neurological Surgery, Hamad General Hospital, Doha, QAT

**Keywords:** aneurysmal subarachnoid haemorrhage, endovascular coiling, hemiparesis, posterior communicating artery aneurysm, true posterior communicating artery aneurysm

## Abstract

Posterior communicating artery (PCOM) aneurysms, even though rare, can lead to severe neurological deficits alongside life-threatening conditions, demanding rapid detection and intervention. We report a case of a 42-year-old male patient, known hypertensive, presenting with symptoms of headache, dizziness, and right-sided weakness. Imaging showed a saccular aneurysm in the left middle cerebral artery (MCA) alongside an acute-subacute subarachnoid hemorrhage. A subsequent computed tomography (CT) angiography established a large PCOM aneurysm. Above all, the aneurysm caused hemiparesis, a comparatively rare manifestation. Treated effectively through a balloon remodeling coiling technique, the patient recovered postoperatively. This case report also highlights the significance of timely recognition and the usefulness of endovascular coiling in treating PCOM aneurysms. Precisely, those patients presenting with atypical symptoms like hemiparesis lay stress on the need for strong clinical vigilance and further research to improve management strategies intended for these rare but critical conditions.

## Introduction

Intracranial aneurysms, predominantly those affecting the posterior communicating artery (PCOM), present a considerable risk in line with their potential for tragic consequences. These aneurysms, yet infrequent, can lead to severe neurological deficits besides life-threatening conditions, emphasizing the critical necessity for rapid detection as well as intervention.

PCOM aneurysms are a rare subtype of intracranial aneurysms, arising from the posterior communicating artery, accounting for merely 1.3% of the entire cases [1]. They characteristically arise from the posterior communicating artery, a crucial vessel within the brain's vascular system. The clinical implication of PCOM aneurysms lies not only in their uncommonness but also in their tendency to produce severe neurological symptoms upon rupture or significant growth.

Intracranial saccular aneurysms, together with PCOM aneurysms, have been identified through radiographic imaging as well as autopsy studies in almost 3.2% of the overall population, primarily affecting individuals about the age of 50 who have no other comorbid conditions [2]. Remarkably, these aneurysms remain more predominant in females, representing 61% of the cases. This gender difference turns out to be even more distinct in individuals above the age of 50, where the female-to-male ratio upsurges to 2:1 or greater.

The development of intracranial aneurysms remains inclined by a combination of genetic in addition to modifiable risk factors. Genetic predispositions consist of hereditary syndromes such as connective tissue disorders (e.g., Ehlers-Danlos syndrome and pseudoxanthoma elasticum), familial aldosteronism type I, and Moyamoya syndrome. Modifiable risk factors that considerably uplift the possibility include cigarette smoking, hypertension, and estrogen deficiency, stressing the importance of lifestyle and hormonal balance in the pathogenesis of these aneurysms [2].

PCOM aneurysms generally manifest with symptoms, for instance severe headache, loss of visual acuity, and cranial nerve deficits, especially third cranial nerve [3]. Regardless of these typical presentations, hemiparesis or partial paralysis on one side of the body is an atypical presentation and has been documented in just seven case reports. This symptom, however rare, highlights the varied and occasionally unpredictable clinical manifestations of PCOM aneurysms.

Knowing the high possibility of rupture related to PCOM aneurysms and the severe consequences that can follow, timely detection and immediate management are imperative [3]. The following case report exemplifies a patient presenting with an acute onset of hemiparesis, eventually identified with a PCOM aneurysm, laying emphasis on the compulsion for sharp clinical attentiveness and rapid therapeutic action in such cases.

## Case presentation

A 42-year-old man arrived at our emergency department, suffering from a headache and dizziness that had remained for two days. It was not relieved despite taking painkillers. He is a known hypertensive and had been diligent with his medication. Up until lately, he was non-compliant with his antihypertensive medications for the past two days. His blood pressure on presentation was 170/100, On physical examination, he was found to be drowsy and unresponsive to verbal commands. Additionally, he showed right-sided weakness, graded as 4/5 strength, as well as right facial palsy, but no additional focal neurological deficits were detected.

**Figure 1:**
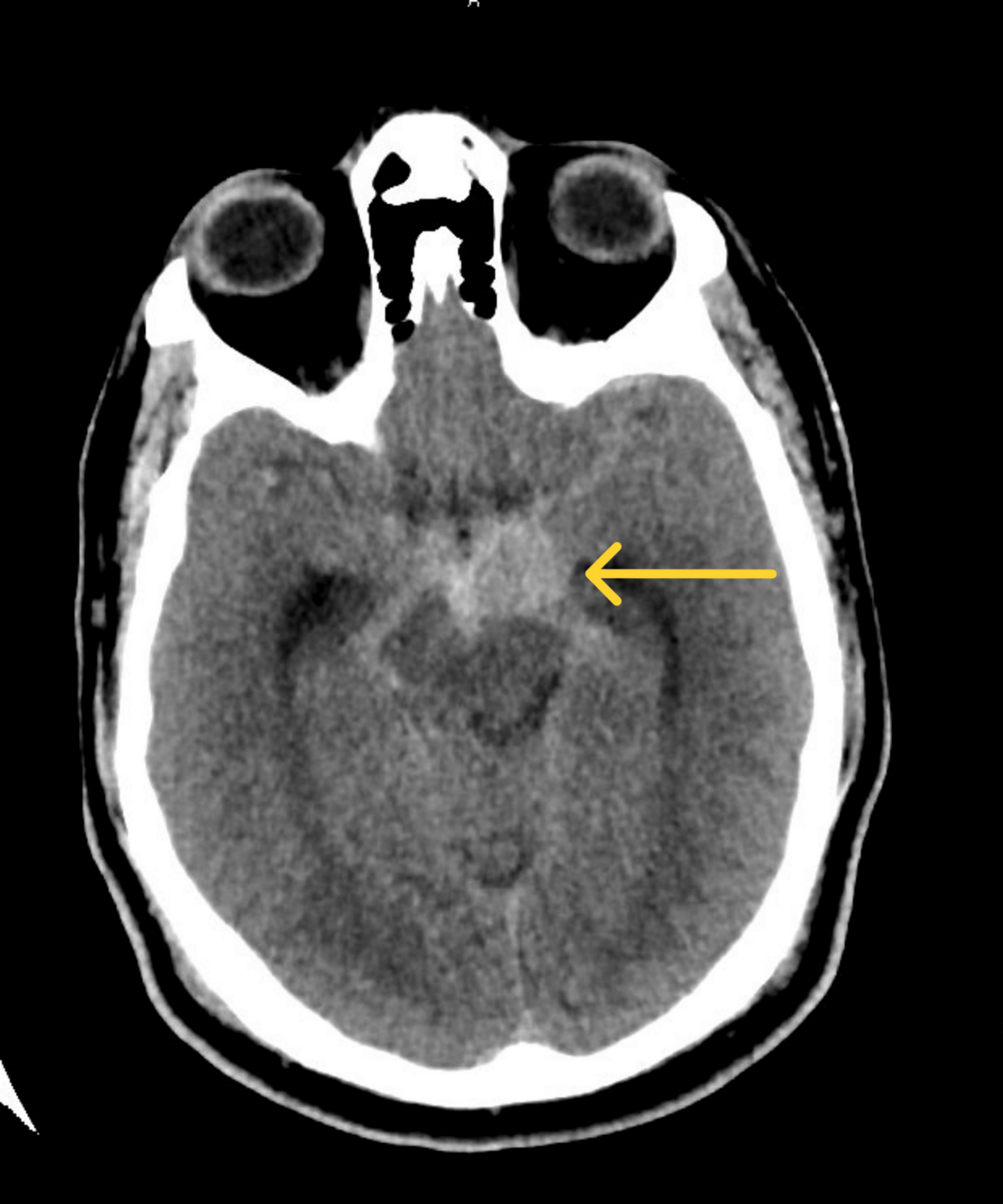
Head CT scan, axial view showing small saccular aneurysm in the M1 segment of the left middle cerebral artery and also an acute-subacute subarachnoid hemorrhage, with modified Fischer grade 1

A CT scan of the head was done to exclude intracranial hemorrhage and stroke. The image showed a small saccular aneurysm in the M1 segment of the left middle cerebral artery (MCA) and also an acute-subacute subarachnoid hemorrhage. There were no infarcts in the midbrain or the cerebral hemisphere (Figure 1).

**Figure 2:**
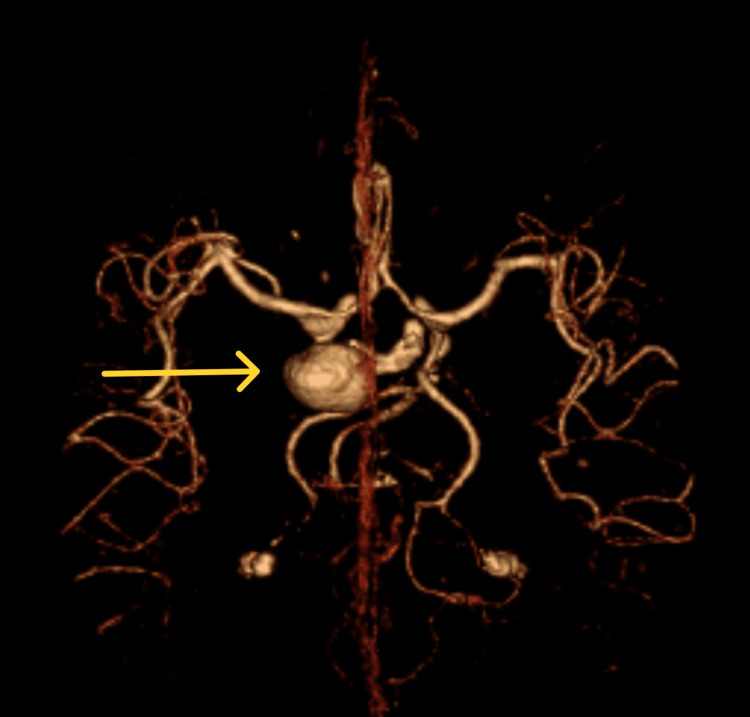
3D Computed tomography angiography, showing large aneurysm in the left posterior communicating artery.

**Figure 3:**
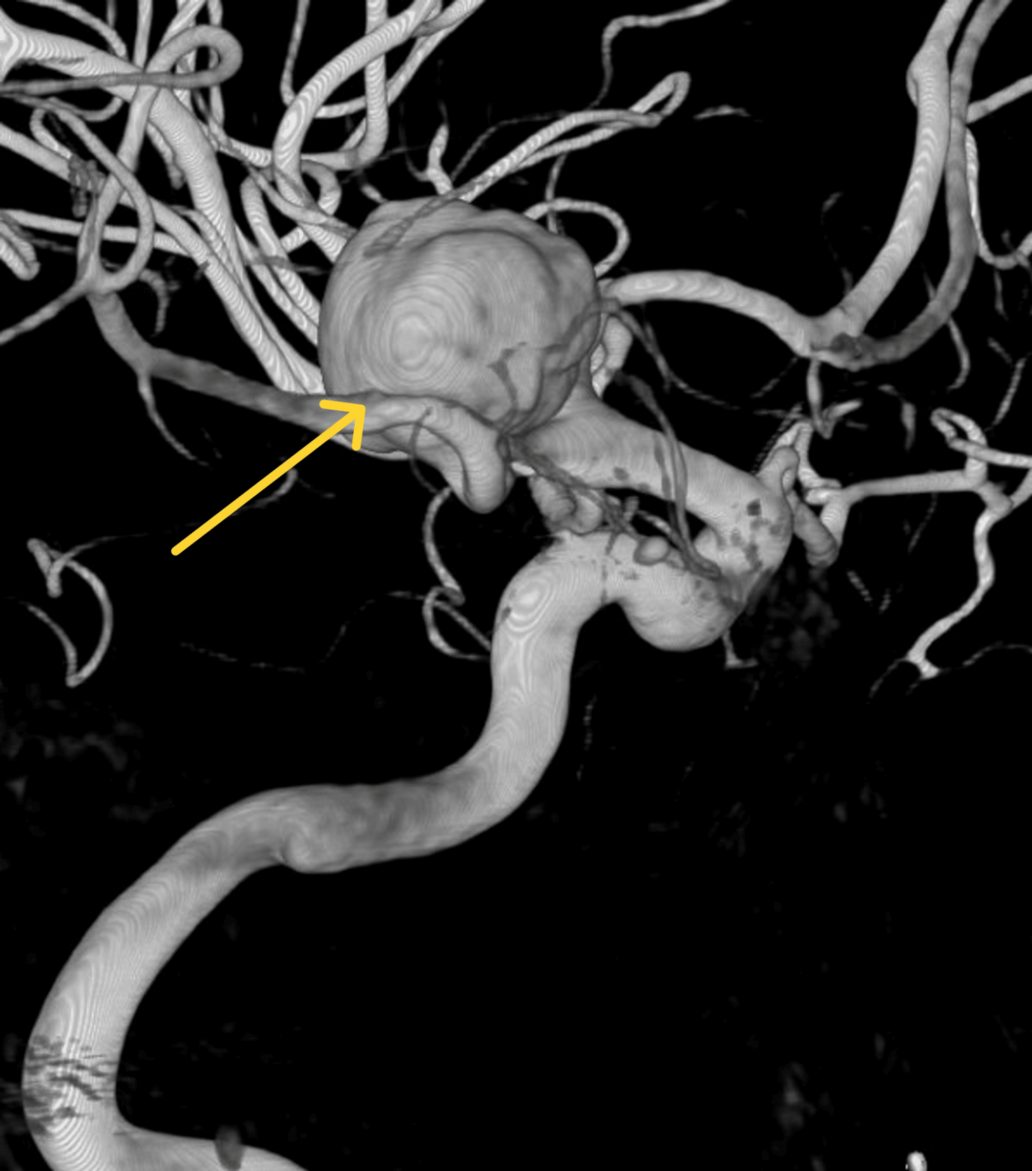
3D Computed tomography angiography, showing large aneurysm in the left posterior communicating artery.

3D CT angiography revealed a large aneurysm in the left posterior communicating artery (Figures 2, 3).

**Figure 4:**
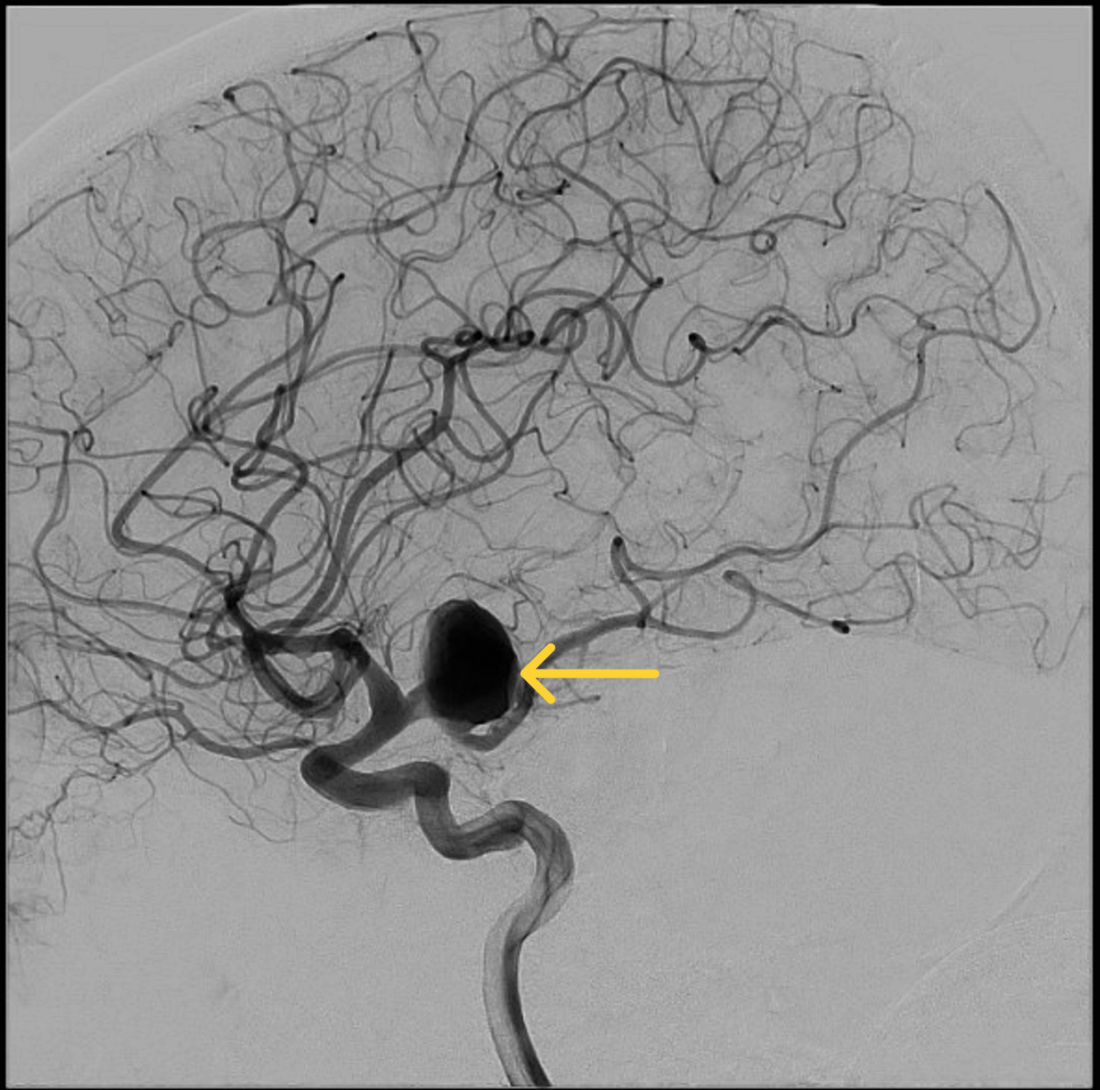
Cerebral angiogram, lateral view showing large aneurysm in the left posterior communicating artery.

**Figure 5:**
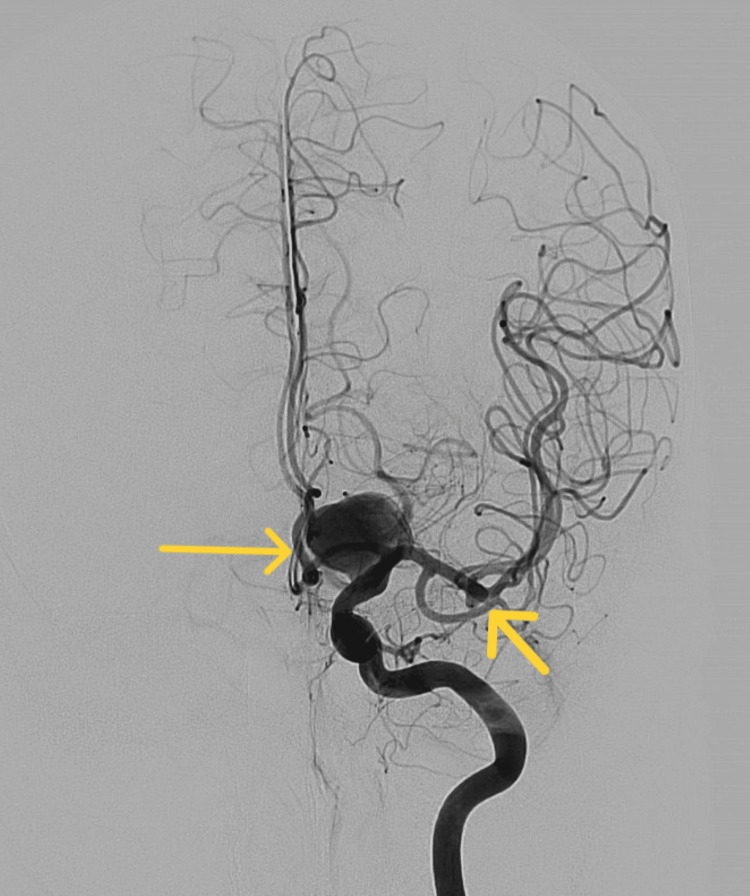
Cerebral angiogram, posterior view showing large aneurysm in the left posterior communicating artery and small aneurysm by the left MCA bifurcation. MCA: middle cerebral artery.

A cerebral angiogram revealed a 20mm aneurysm with 15mm neck in the posterior communicating artery (Figure 4). This aneurysm was treated through a balloon remodelling coiling technique under general anesthesia. During the procedure, another small aneurysm was found by the left MCA bifurcation (Figure 5).

**Figure 6:**
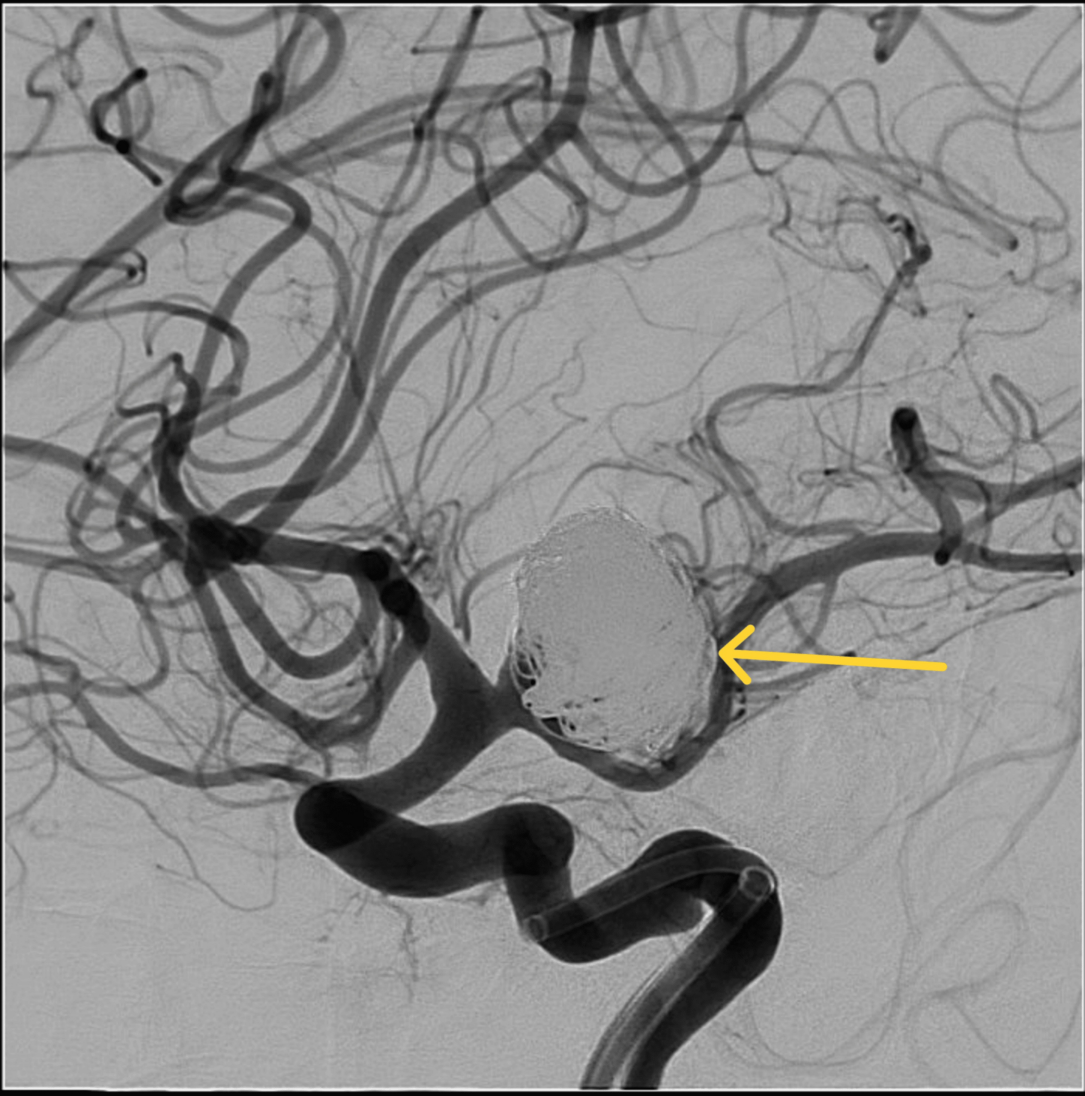
Post-coiling cerebral angiogram showing occlusion of the aneurysm, with small residual neck

For the time being, it was not the main source of hemorrhage. It was left untreated and planned for impending elective management. A control angiogram confirmed the complete obstruction of the aneurysm (Figure 6).

At that time, the patient was moved to the surgical intensive care unit (SICU) after the procedure, where he remained sedated and intubated. His stay in the SICU was uneventful; above and beyond, he completely improved from his pre-operative weakness. He was then moved to the ward intended for further observation and care. Succeeding his hospital stay, the patient was followed up in the outpatient department; the patient will undergo further follow-up with repeat imaging to check any possibility of regrowth.

## Discussion

Intracranial aneurysms, mostly those concerning the PCOM, characterize a subset of cerebrovascular pathologies. These abnormalities require a complete understanding due to their probability of serious neurological sequelae. This discussion addresses the epidemiology, clinical manifestations, diagnostic strategies, and treatment modalities, in addition to case studies that are relevant to PCOM aneurysms. 

Epidemiology and risk factors

PCOM aneurysms are relatively uncommon although they display demographic tendencies as well as possible genetic tendencies. Studies point toward an inclination for males above 50 years of age, constant with broader trends in intracranial saccular aneurysms [4]. These aneurysms may also be influenced by genetic factors, including connective tissue disorders and familial clustering, which contribute to an individual’s susceptibility. Broadly speaking, age, sex, and genetic predispositions remain key factors in aneurysm formation [5].

Clinical presentation

The clinical presentation of PCOM aneurysms includes a range of neurological deficits, reflecting the varied anatomical territories supplied by the artery. Symptoms generally consist of acute onset headache, unilateral hemiparesis, visual disturbances, and, in unusual cases, oculomotor nerve palsies [6]. Hemiparesis, which in our case is to due to a direct compression of the aneurysm and the hematoma on the cerebral peduncle, however not as frequent, emphasizes the potential for significant motor damage.

Diagnosis

True PCOM aneurysm embodies the rarest form of intracranial aneurysm. The diagnosis of aneurysms characteristically depends on several imaging modalities, for example, CT angiography, magnetic resonance angiography (MRA), or digital subtraction angiography. Over the past two decades, advancements in these imaging techniques have noticeably enhanced the accuracy and effectiveness of aneurysm detection [2]. These techniques give an accurate visualization of the vascular architecture, allowing clinicians to distinguish aneurysmal morphology, size, and location. Improved diagnostic capabilities over recent years have helped in better detection rates, vital for timely intervention.

PCOM artery aneurysms include a range of locations, together with junctional aneurysms involving the internal carotid artery (ICA) and PCOM, posterior wall aneurysms of the ICA not linked towards the PCOM origin, junctional aneurysms of the PCOM and posterior cerebral artery (PCA), and least commonly, those originating 2-3mm posteriorly from the PCOM itself [7].

The PCOM artery serves as the origin of several important divisions, providing serious anatomical structures, for example the optic chiasm, oculomotor nerve, mammillary body, tuber cinereum, cerebral crura, ventral thalamus, and rostral portion of the caudate nucleus [1]. Protection of these branches arises as a critical determining factor influencing patient outcomes. The widespread branching network originating from the PCOM contributes to the varied clinical presentations witnessed in PCOM aneurysms. To the best of our knowledge, amongst known cases of PCOM aneurysms, the display of hemiparesis has been reported in merely seven additional cases, underlining its comparatively uncommon incidence in this background.

Treatment options

The management plans for PCOM aneurysms are centred upon numerous factors, together with aneurysm size, location, patient's clinical status, and comorbidities. Endovascular coiling and surgical clipping signify the main therapeutic modalities, individually with separate advantages and considerations [8]. Surgical clipping usually served as the cornerstone treatment, involving careful dissection and protection of vital arterial branches. Endovascular coiling has arisen as an alternative way of offering minimally invasive aneurysm obliteration.

Surgical clipping

Throughout the surgical procedure for PCOM aneurysms, the initial step includes carefully splitting the Sylvian fissure, in addition to then locating it back alongside the internal carotid artery (ICA) to find the origin of the PCOM [9]. The aforementioned is crucial, as surgeons must have a profound understanding of the anatomy for the reason that the PCOM supplies several main branches. Keeping these divisions unharmed is vital for the patient's recovery. Even though the PCOM is smaller than normal, it is crucial to preserve it beside with the 4-14 thalamoperforating arteries. Surgeons correspondingly must be watchful of the nearby oculomotor nerve, which goes together with the PCOM, to evade causing injury. In a single case described by Nagatani et al., they successfully performed microsurgical clipping on a patient with a PCOM aneurysm, resolving hemiparesis but then again leaving them with persistent oculomotor palsy [1].

Microsurgical clipping remains a delicate procedure frequently necessitating a pterional approach to reach the aneurysm. The objective remains to cautiously clip the aneurysm even though safeguarding the PCOM and its associated arteries remains intact though it is essential to acknowledge that surgery can occasionally lead to permanent neurological issues. This underlines the necessity for surgeons to be extremely skillful and careful in selecting which patients remain appropriate for this kind of intervention.

Endovascular coiling

Endovascular coiling has turned out to be a popular, less invasive substitute to surgical clipping aimed at treating PCOM aneurysms. This procedure consists of threading micro-catheters into the aneurysm and then placing platinum coils inside to promote clotting and efficient closure of the aneurysm [10]. Even if endovascular coiling by and large yields good results, it needs specialized skills in neuro-interventional methods as it also comes with probable risks such as coil migration and the possibility of the aneurysm reopening. Nonetheless, reports on the efficacy of this modality to treat PCOM aneurysms are limited due to its rarity of occurrence [7].

In the case presented in this report, the patient presented with acute-onset hemiparesis and remained to have a PCOM aneurysm on CT angiography. The patient underwent a successful endovascular coiling of the aneurysm with a good postoperative outcome.

Case studies

Several case reports have described the presentation, treatment, and outcomes of patients with PCOM aneurysms. In an analysis of eight cases, including our case, the majority were males above the age of 50 who presented with headaches besides unilateral hemiparesis. Patients underwent various treatments, comprising surgical clipping, endovascular coiling, and mechanical thrombectomy with stenting. Most patients experienced substantial improvement in their neurological status after treatment, except for a single case that described persistent oculomotor palsy succeeding surgical clipping.

**Table 1:** Case Studies of PCOM Aneurysms: Clinical Presentations, Treatment, and Outcomes PCOM: Posterior communicating artery.

References	Age, sex	Initial Presentation	Treatment	Outcome
Abbasher et al. (this case report)	42, M	Headache and dizziness for two days. Drowsy and unresponsive to verbal commands. Right-sided weakness, 4/5 strength. Right facial palsy.	Balloon remodeling coiling technique under general anesthesia.	Completely improved from his pre-operative weakness
Nagatani et al. (2012) [1]	59, F	Severe headache, associated with vomiting, left hemiparesis, and right oculomotor nerve palsy.	Right fronto-temporal craniotomy and intra-dural anterior clinoidectomy + clipping	Patient was discharged. Resolved hemiparesis. Persistent right oculomotor palsy
Munnariz et al. (2014) [7]	53, M	Sudden onset of right hemiparesis and aphasia	Endovascular treatment with coiling	Improvement of neurological status, discharged
Kocak et al. (2013) [11]	52, M	Acute consciousness disturbances and right-sided motor weakness	Endovascular occlusion of the aneurysm with coils	Followed as an outpatient for 30 months; neurological symptoms resolved
	60, M	Mild headache and left-sided numbness lasting for a few days	Flow diverter treatment	Follow-up; after one year, patient’s neurological symptoms have resolved
Imai (2001) [12]	47, M	Semicoma and left hemiparesis	No intervention due to patient’s critical neurological state	Died of rebleeding
Otsuji et al. (2017) [13]	87, F	Disturbance of consciousness and left hemiparesis	Mechanical thrombectomy in combination with a stent retriever and the Penumbra system	Fully recovered from left hemiparesis and impaired consciousness, discharged
Hayashi et al. (1997) [14]	65, M	Sudden disturbance of consciousness and left hemiparesis	Aneurysm was clipped via pterional approach	Recovered and discharged

## Conclusions

True PCOM aneurysms are an infrequent form of intracranial aneurysms that can exist with a diversity of symptoms, including headache, altered mental status, and focal neurological deficits. Accurate diagnosis depends on advanced imaging techniques, and treatment choices include surgical clipping and endovascular coiling. Although both approaches have been presented to be effective, vigilant consideration of the aneurysm's location and association to nearby structures is vital for augmenting results. Additional research is required to establish best practices for the management of this rare but challenging condition.
